# New northernmost distribution records of the Eastern South Pacific southern right whale (*Eubalaena australis*), including the first cases from Ecuador and northern Peru

**DOI:** 10.1371/journal.pone.0312528

**Published:** 2024-11-11

**Authors:** Cristina Castro Ayala, Ana M. García-Cegarra, Piero Uceda-Vega, Luis Aguilar, Shaleyla Kelez, Susannah J. Buchan, Fernando Félix, Stephanie H. Stack, Koen Van Waerebeek

**Affiliations:** 1 Pacific Whale Foundation-Ecuador, Malecón Julio Izurieta, Puerto López, Manabí, Ecuador; 2 Facultad de Ciencias del Mar y Recursos Biológicos, Instituto de Ciencias Naturales Alexander von Humboldt, Universidad de Antofagasta, Antofagasta, Chile; 3 Marine Megafauna Research Laboratory (CETALAB), Antofagasta, Chile; 4 World Wildlife Foundation-WWF Peru, Lima, Peru; 5 Ecoceanica, Lima, Peru; 6 Center for Oceanographic Research COPAS COASTAL, Universidad de Concepción, Casilla, Concepción, Región del Bio, Chile; 7 Facultad de Ciencias Naturales y Oceanográficas, Departamento de Oceanografía, Universidad de Concepción, Casilla, Concepción, Región del Bio, Chile; 8 Centro de Estudios Avanzados en Zonas Áridas (CEAZA), Raul Bitran, La Serena, Chile; 9 Pontificia Universidad Católica del Ecuador (PUCE), Quito, Ecuador; 10 Museo de Ballenas, Salinas, Ecuador; 11 Pacific Whale Foundation, Wailuku, HI, United States of America; 12 Southern Ocean Persistent Organic Pollutants Program, Centre for Planetary Health and Food Security, Griffith University, QLD, Australia; 13 Peruvian Centre for Cetacean Research, Centro Peruano de Estudios Cetológicos (CEPEC), Museo de Delfines, Lima, Peru; 14 ProDelphinus, Miraflores, Lima, Peru; DePaul University, UNITED STATES OF AMERICA

## Abstract

The Eastern South Pacific Right Whale (SRW) (*Eubalaena australis*) population has gained interest due to its Critically Endangered conservation status. So far, this population has been confirmed only along the coasts of Chile (18°20’S to 56°30’S) and from southern to central Peru (17°38’S to 12°11’S). Recent records have extended the species’ known range, highlighting its geographic distribution, now reaching 1500 km north. Here, we report six recent records, consisting of five sightings and one stranding, that expand the documented range to northern Ecuador (0.6°N). The northern extension of the population may be associated with the unusual three-year-long cold phase (La Niña) of the El Niño Southern Oscillation (ENSO) in the eastern South Pacific, population expansion, movement and re-distribution of the species, increased monitoring effort, or a combination of these factors. These observations raise hope for the Critically Endangered SRW population, as the occurrence of mother-calf pairs may indicate a potential for population recovery. Nevertheless, these findings intensify concerns for what is still the least abundant SRW population, underscoring the urgency for more targeted research and conservation measures.

## Introduction

Southern right whales (SRW) (*Eubalaena australis*) from the Eastern South Pacific (ESP), previously referred to as the Chile-Peru population (CPe) [1], move seasonally from feeding areas located in high-latitude sub-Antarctic waters to breeding areas located in mid-latitude waters [2]. This species mainly inhabits coastal waters, although they may travel offshore during their migration [3]. The ESP population was hunted extensively in the 18th and 19th centuries by American and French whaling fleets and in the 20th century by Chilean whalers [4]. Past overexploitation and their coastal distribution have rendered them highly vulnerable to anthropogenic impacts, resulting in their near extinction. [5]. The ESP population has shown significantly reduced signs of recovery compared to other SRW populations in the Southern Hemisphere, *i*.*e*., Argentina, South Africa, Australia, and New Zealand [6–9]. It is one of two IUCN-defined ‘Critically Endangered’ whale populations worldwide, with fewer than 50 mature individuals estimated [7,10]. The status of SRW in the ESP was assessed by the International Whaling Commission (IWC) Scientific Committee, reviewing 79 sightings collected between 1975 and 2010 in southern Peru and Chile, with records documented between June and November [5,8,11–15]. The known distribution of the species extends from Chorrillos, Lima, central Peru (12° 11’S) south to the Gulf of Penas, southern Chile (47° 58’S) [13,14,16–18]. Antofagasta (23° 39’S), northern Chile, has been described as a potential nursery area [5]. A record of two ‘like SRWs’ off Punta Sal (03°59’S), Tumbes, in northern Peru in early August 2005, supported by low-resolution photographs [14], strongly suggested that Lima is not the northern distribution boundary for ESP SRW.

Due to this population’s status and the urgent need for conservation measures, a Conservation Management Plan (CMP) for the ESP SRW was developed and endorsed by the International Whaling Commission in 2012 [19]. The Governments of Chile and Peru supported the CMP due to the lack of signs of population recovery. The CMP aims to guide and encourage various stakeholders (i.e., government, industry, coastal communities, whale watching operators, fishermen, scientists, and international partners) to take steps towards the recovery of this population to levels that will allow it to withstand both environmental and anthropogenic impacts, ensuring its long-term survival [19,20]. The CMP acknowledges the need to generate baseline data on demography, distribution, stock structure, and threats to design mitigation strategies. Advances have been reported since its implementation, including increased monitoring efforts through passive acoustics and response to entangled whales, photo-identification, and a citizen science strategy [21]. Due to the extensive distribution range of this population in the Southeast Pacific, coordinated efforts through an internationally adopted management framework is the way forward to support the population recovery.

We present new SRW records with accompanying photographic evidence along the west coast of South America. A preliminary version of this report was presented to the Scientific Committee of the International Whaling Commission [22] and here we expand upon, include new records and contextualize these findings.

## Materials and methods

### Study area

The investigated area includes the coasts of Ecuador, Peru and Chile, a region strongly influenced by the Humboldt Current System that originates around 45°S in southern Chile and extends 4,500 km north toward the central coast of Ecuador, before diverting west to join the South Equatorial Current. This is one of the most productive marine ecosystems in the world due to the intense upwelling of deep, cold, nutrient-rich waters, leading to increased primary productivity [23]. Important fisheries are conducted in the region, including for the Peruvian anchovy (*Engraulis ringens*), Pacific sardines (*Sardinops sagax*), Humboldt squid (*Dosidicus gigas*) and different species of tuna and sharks, among others. In northern Chile, the rich primary productivity favors an abundance of red squat lobster (*Pleuroncodes monodon*) and krill (*Euphausia mucronata*) [24,25]. The latter and anchovy are the main prey for several of the 19 cetacean species known to occur in the region [5,26].

### Data collection

The extreme low abundance of SRWs in the study area renders dedicated survey efforts for this species impractical. Thus, all records have been obtained opportunistically or as part of other ongoing projects or citizen science reports. In Chile, SRW sightings were reported by fishermen and local inhabitants, with information typically shared through social media platforms as part of a citizen science initiative. In the Antofagasta area, SRWs were sighted during systematic surveys for cetaceans as part of a long-term monitoring project [5,26,51]. In northern Peru, a SRW was sighted during systematic boat surveys for humpback whales, studying behavioral responses to whale-watching boats off El Ñuro (4°12’S; 81°9`W) [27,28]. In Ecuador, sighting records included photographs and videos taken by crews of commercial whale-watching vessels operating in the Machalilla National Park, where long-term research and citizen science projects studying humpback whales (*Megaptera novaeangliae*) have been conducted for three decades [29–31]. Researchers confirmed and documented all sightings through videos and photographs. Additionally, colleagues studying cetaceans in the ESP region were consulted to inquire about potential missed SRW records. In the case of the only stranding, one of the authors (C.C.) was informed by a park ranger who is a member of the Ecuador Stranding Network and attended the event to take tissue samples, measurements, and photographs. Identification as SRW was based on descriptions of their distinct morphological characters, particularly the absence of a dorsal fin, the prominent arch of the mouth, and the callosities on the sides and head of the whale [4,6,32]. The stranding event was assisted via email by scientists from the IWC expert advisory panel on strandings and other scientific experts. There exists no public central database specifically for SRW records in the ESP; instead, information was collated from various initiatives, including systematic cetacean surveys, citizen science reports.

In Peru, aerial imagery was collected using a DJI Phantom 4 Pro drone (DJI Innovation, Shenzhen, PRC) equipped with a gimbaled camera (1” CMOS, 20 M effective pixels). A high-quality video of the individuals was obtained to identify the mother and calf as SRW based on the presence of callosities and to calculate their body lengths relative to the known length of the research vessel according to methodology by [33]. Photo identification of SRW head callosities was collected by drone. Potential matches were checked for with the SRW photo catalogue for the ESP, updated with sightings provided in this study [5].

Acoustic recordings were made with an H2a-XLR omnidirectional hydrophone (sensitivity of 180 dBV/uPa +4 dB, from 20 Hz to 100 kHz) and Tascam DR-40 tape recorder (WAV files, 16-bit, 44.1 kHz). The location of the recording was in a water depth of 20 m and the hydrophone was deployed 10 m below the surface. The recording period was the day of observation of the SRW pair swimming nearby the research vessel and the hydrophone was deployed at a distance of approximately 50–80 m from the mother-calf pair.

Acoustic data were recorded continuously at 48 kHz and analyzed in Raven Pro 2.0 [34] as spectrograms (Hann Window) by an experienced bio-acoustician (SJB) to manually annotate SRW calls based on their frequency and duration [sensu 30]. Seven call types were checked (upcall, down-call, down-upcall, tonal constant, tonal variable, hybrid and pulsive), all below approximately 500 Hz [35]. For each potential call, the peak frequency, beginning and end frequency, and the duration of the call were measured using the Selection Table function in Raven Pro 2.0. Average peak frequency, beginning and end frequency, and duration (± SD) were registered for comparison to calls reported by [35].

### Environmental data

Whale records were correlated with environmental variables such as depth and the Oceanic Niño Index (ONI). ONI is a measure of the El Niño-Southern Oscillation strength developed by the NOAA Climatic Prediction Center (https://www.cpc.ncep.noaa.gov/products/analysis_monitoring/ensostuff/ONI_v5.php). It is calculated as a 3-month running average of Sea Surface Temperature (SST) anomalies (SSTA) in the Niño 3.4 region (120° –170°W, 5°N –5°S) of the Equatorial Pacific Ocean (EPO).

## Results

### The records

From the compiled data, we found six new records of SRW in 2022 and 2023 that have not been previously reported, including five live sightings and one stranding (Table 1). All sightings consisted of mothers with a dependent calf. Most records were made within Marine Protected Areas (MPA), namely Machalilla National Park and San Francisco-Galera Marine Reserve in Ecuador, and La Rinconada and La Portada Marine Reserves in northern Chile in August 2022. Since tourism and research activities are both concentrated within these MPAs, records are biased efforts towards these regions. Below we describe the six new SRW records in some detail.

**Table 1:** Chronologic summary of six new SRW records of the ESP population in 2022–2023, including 5 sightings of mother-calf pairs (# 1–5), and one stranding of a calf (# 6). Two environmental parameters (depth and ONI Index, see text) are included. Warm and cold periods based on a threshold of +/- 0.5°C for the ONI.

Record	Date	Location/Country	Position	Depth^a^ (m)	ONI^b^
**1**	8-Aug-22	El Ñuro, Los Organos, Perú	4° 12’S, 81° 11’W	100	**-0.8**
**2**	16-Aug-22	La Rinconada, Antofagasta, Chile	23° 28’S, 70° 30’W	500	**-0.9**
**3**	31-Aug-22	Pisagua, Iquique, Chile	19° 34’S, 70° 12’W	1000	**-1**
**4**	11-Sept-22	Salango Is., Puerto Lopez, Manabi, Ecuador	1° 35’S, 80° 53’W	20–50	**-1**
**5**	21-Aug-23	Sucre Is., Machalilla, Manabi, Ecuador	1° 28’S, 80° 47’W	10–20	**1.3**
**6**	28-Aug-23	Tongorachi, Muisne, Esmeraldas, Ecuador	0° 40’N, 80° 5’W	N/A	**1.3**

^a^ Depth data from GEBCO: https://www.gebco.net/data_and_products/gridded_bathymetry_data/.

^b^ ONI index from: Climate Prediction Center - ONI (noaa.gov).

### Record # 1

**Fig 1:**
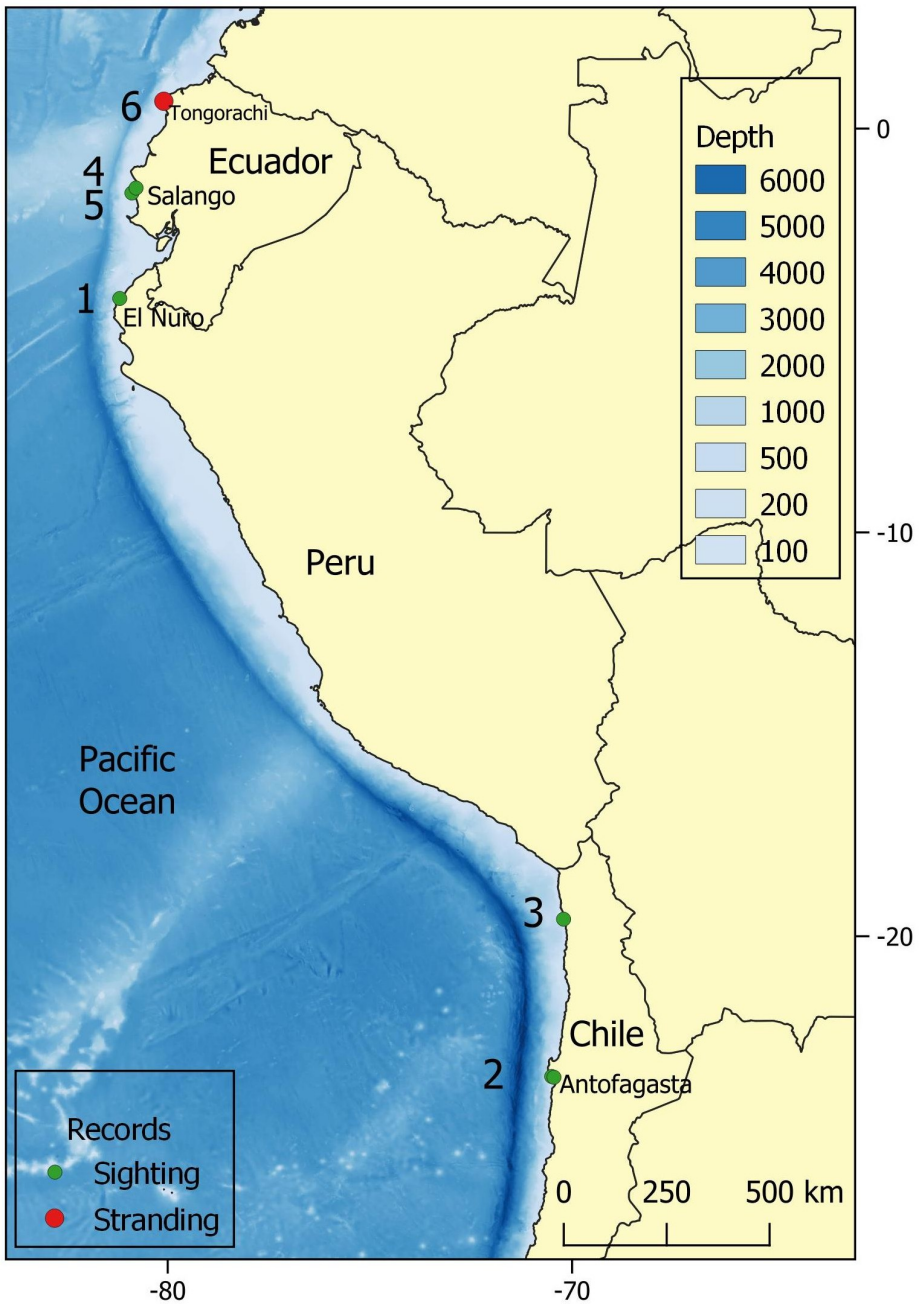
The study area and location of southern right whale observations. Mother-calf pairs sighted off 1) El Ñuro, northern Peru; 2) the Antofagasta area, northern Chile, from 15 to 18 August, 2022; 3) Pisagua, Iquique Region, northern Chile; 4) Salango Island, Ecuador; 5) Sucre Islet, Machalilla, Ecuador; and 6) stranding of a calf in Tongorachi, Esmeraldas, northern Ecuador. The data used to produce this map were all from public sources, the national boundary were obtained from Natural Earth (http://www.naturalearthdata.com.

**Fig 2:**
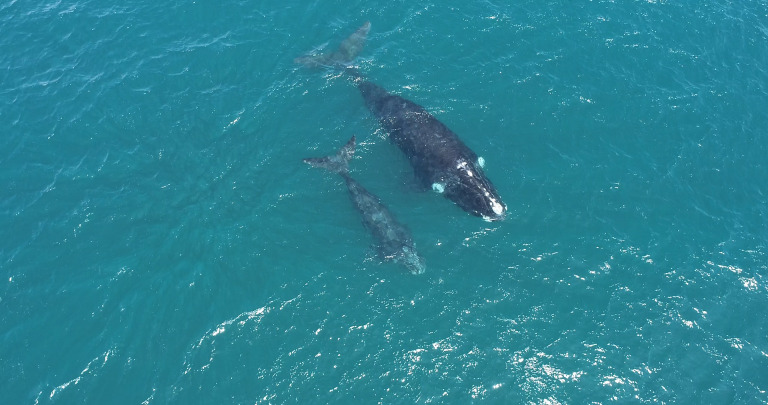
Aerial image of a mother and calf SRW observed off El Ñuro, Piura, northern Peru, on 8 August 2022.

On 8 August 2022, a SRW mother-calf pair was observed off El Ñuro, Piura, northern Peru (Table 1, Fig 1) (Beaufort sea state = 1; visibility >10 km). The whales were swimming close to shore in a northward direction. During a 12-minute drone flight, high-quality video of the individuals was captured (Fig 2). The images obtained facilitated the photo-identification of the mother-calf pair. The body lengths of the mother and calf were estimated to be 12.2 meters (SD ± 0.7) and 6.4 meters, respectively. A comparison of the head callosities of both the mother and calf was conducted among available photo-identification catalogs of the ESP population [5], and no matches were found.

**Fig 3:**
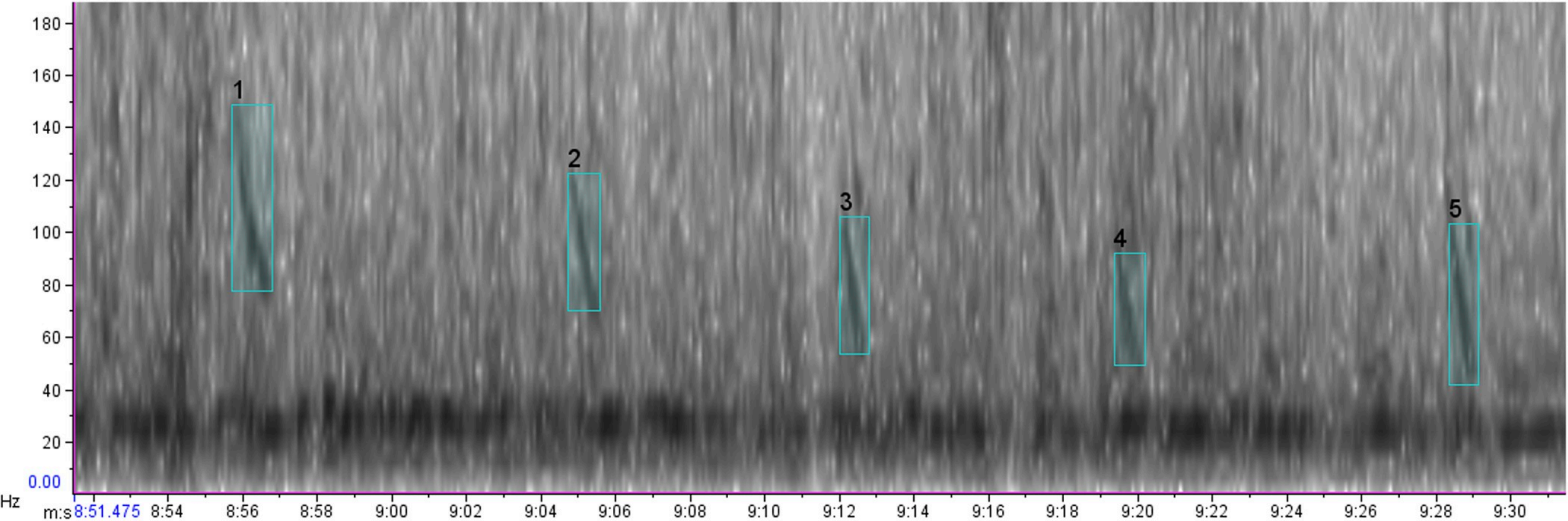
Spectrogram of possible right whale down-calls off El Ñuro, Piura: Highlighted in light blue (Hann window; FFT = 8192 samples; 50% overlap; page duration = 40 s).

Acoustic data from the observed mother-calf pair were recorded at location 4.19°S, 81.16°W for 12.5 minutes, within 50–80 m distance from the target animals (Fig 3). Only nine faint, possible right whale down-calls were identified in the spectrogram (FFT = 8192 samples; 50% overlap; page duration = 40 s) (Table 2, Fig 3).

**Table 2:** Frequency and duration characteristics of possible right whale down-calls, recorded off El Ñuro, Piura, using metric tools in Raven Pro 2.0 and comparison with calls reported by [35].

	Sample size	Start frequency (Hz) (± SD)	End frequency (Hz) (± SD)	Peak frequency (Hz) (± SD	Duration (s) (± SD)
**Possible downcalls [this study]**	9	112 ± 16.4	51.82 ± 13	75.53 ±15.9	0.83 ± 0.1
**Downcalls [35]**	184	163 ± 66	100 ± 57	128 ± 61	0.6 ± 0.2

### Record # 2

**Fig 4:**
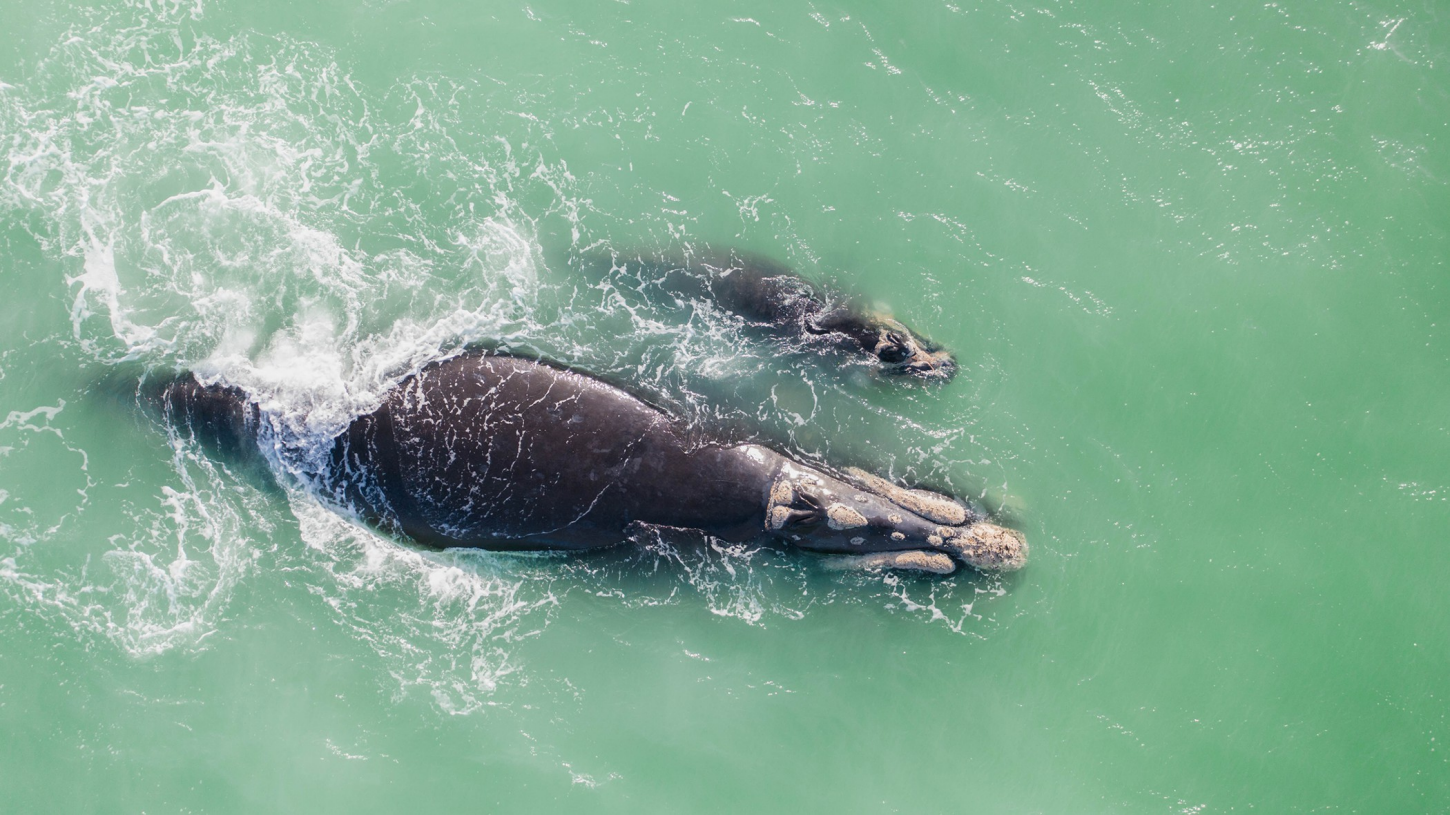
Aerial view of a mother-calf pair SRW observed in La Rinconada Marine Protected Area, Antofagasta Region, on 16 August 2022.

On 16 August 2022, one of the authors (A.G.C.) surveyed the Rinconada Marine Protected Area, Antofagasta Region, northern Chile and found a mother-calf pair (Table 1). A drone was flown for 10 min to record the behavior of the whales and collect an identification image (Fig 4). The whales were observed swimming slowly near the shore and were photo-identified by their head callosities. Due to the turbidity of the seawater, it was not possible to estimate the size of this pair (Fig 4). The identification images were compared with previously cataloged individuals from northern Chile [5] using aerial images of the mother’s head callosities, and no matches with other animals were found. One day earlier, a local fisherman had also reported the presence of a SRW mother-calf pair in that precise area, however, no evidence is available to verify this report. Similarly, a mother-calf pair, presumably the same individuals, were reported in the following two days at La Portada Natural Reserve by locals, some 8 km away from the area where record # 2 was made. However, no imagery was available to confirm the species.

### Record # 3

**Fig 5:**
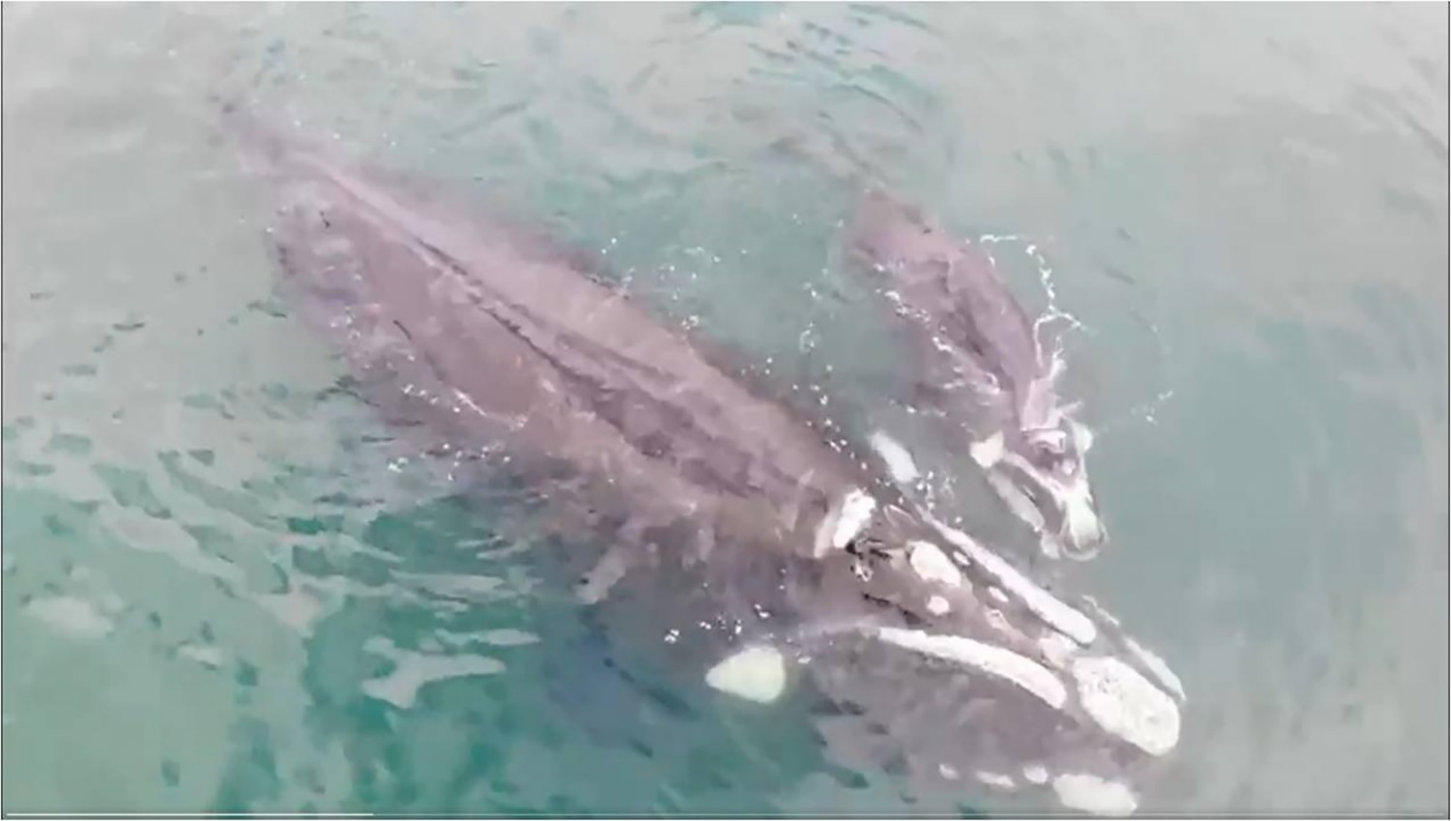
Mother-calf pair SRW observed in Pisagua, Iquique Region, northern Chile, on 31 August 2022.

On 31 August 2022, the Chilean Navy (Dirección General del Territorio Marítimo y de Marina Mercante, Armada de Chile), during coastal navigation in Pisagua, Iquique Region, northern Chile, reported a SRW mother-calf pair. The whales were recorded by drone while swimming close to the shore (Table 1, Fig 5). The images obtained allowed the photo-identification of the pair, and it was determined from the head callosities that they were different from the whales observed in Antofagasta between 15 and 18 August.

### Record #4

**Fig 6:**
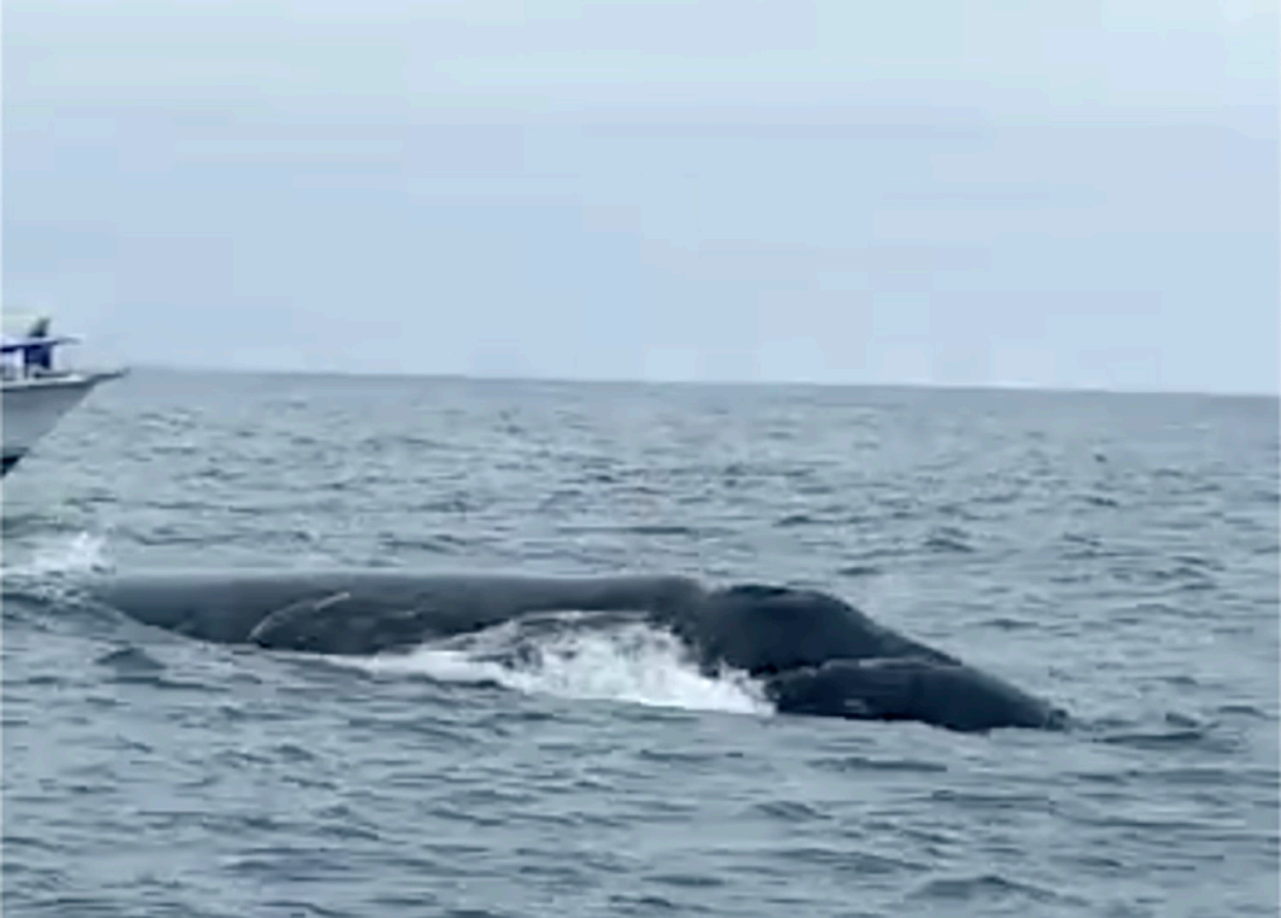
Southern right whale mother and calf observed off Salango Island, Ecuador, on 11 September 2022. Photograph extracted from the S1 Video.

On 11 September 2022, a SRW mother-calf pair was documented east of Salango Island, Machalilla National Park, Ecuador (Table 1, Figs 1 and 6, S1 Video). The sighting lasted 40 minutes and was made by four commercial whale-watching vessels. According to one crew’s report, initially the SRW was resting at the surface. However, upon the approach of the vessels, the whales started zigzagging and headed offshore with increased speed, which was interpreted as an avoidance reaction. The calf swam close to the hind flanks of the mother. At one point, the calf lifted its head and pectoral fin out of the water. The size of the adult whale was estimated at 12–14 m, gauged relative to the size of nearby vessels.

### Record # 5

**Fig 7:**
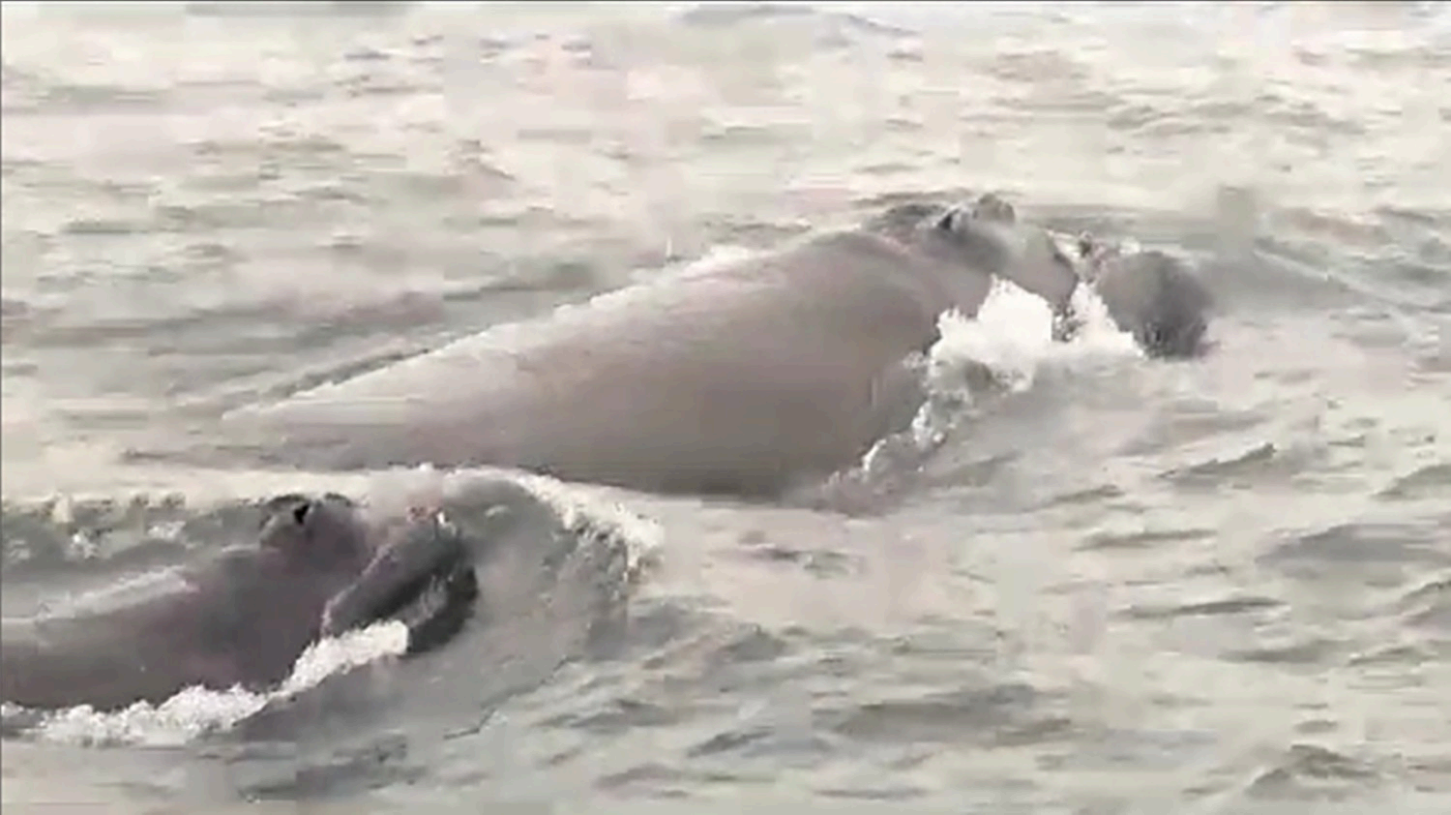
Observation of a mother and calf southern right whale off Sucre Islet, Ecuador, on 21 August 2023. Photograph extracted from the S2 Video.

On 21 August 2023, a mother-calf pair was documented off Sucre Islet, Machalilla National Park, Ecuador (Table 1, Fig 1, S2 Video). The sighting lasted 10 minutes and was made by a commercial whale watching tour, who observed the whales at a distance of approximately 10 m. The crew reported approaching the whale upon noticing it lacked a dorsal fin; the adult whale displayed a pattern of regular surfacing while moving towards Sucre Islet in a westward direction. As the boat approached, the whales increased their breathing rate. The length of the adult was roughly estimated at 14 m when compared to the boat. The crew’s extensive experience with cetaceans lends credence to this information. This record is supported by videos and photographs (Fig 7 and S2 Video).

### Record # 6

On 28 August 2023, a recently deceased SRW calf stranded on Tongorachi Beach in Muisne, within the San Francisco-Galera Marine Reserve, Province of Esmeraldas in Ecuador (Table 1, Fig 1). According to reports from park rangers and community residents, the calf was already deceased when it stranded on the beach. An external examination and a partial necropsy were conducted on 30 August 2023, and showed that the carcass had begun decomposing, with the body slightly bloated and potentially accumulating gases internally, and a carcass condition between 2 and 3 (i.e., 2 = freshly dead; 3 = initial decomposition), according to the criteria defined by [36].

**Fig 8:**
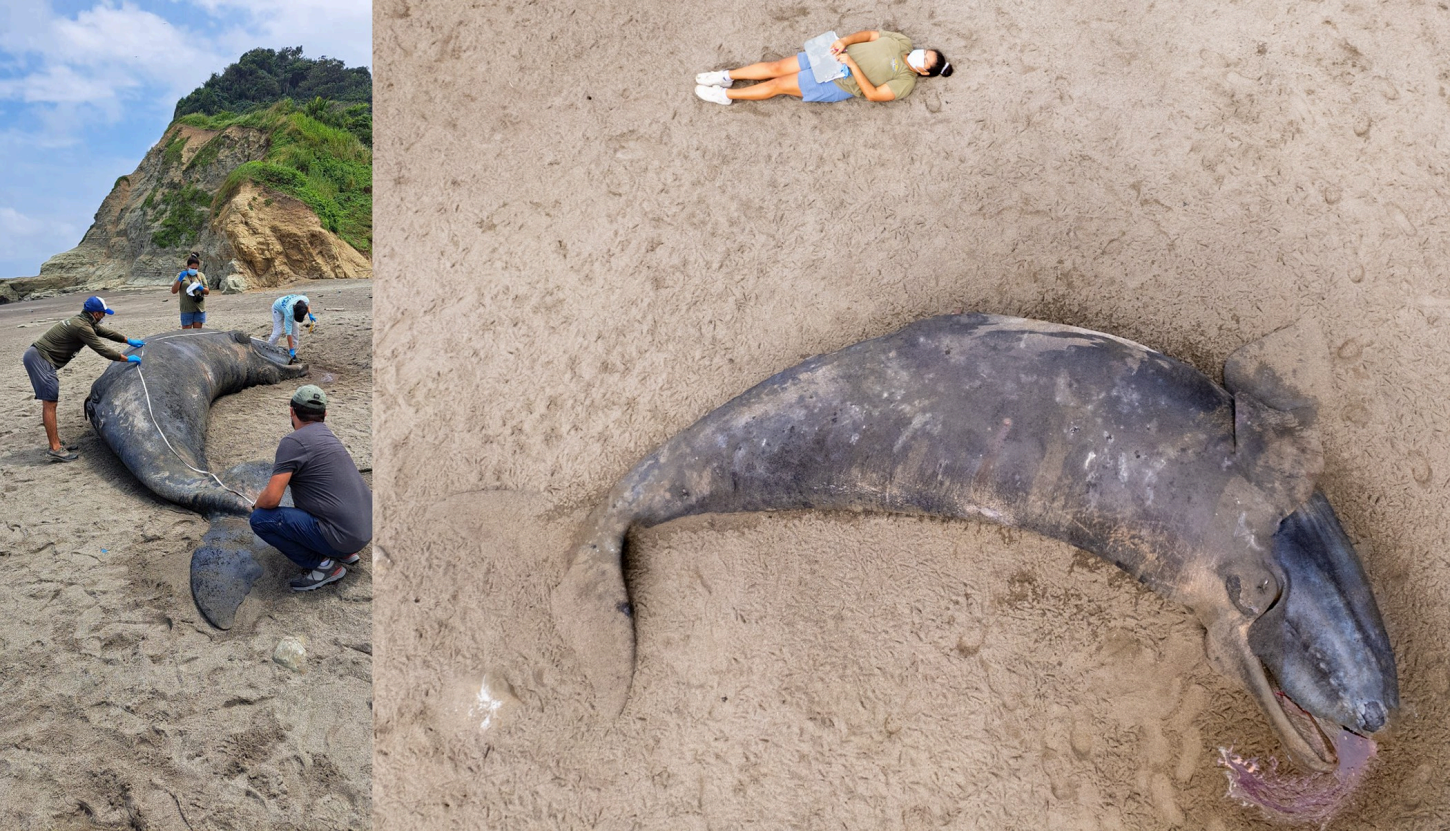
Measuring the stranded right whale specimen along the body’s center (left) and the aerial photograph used to estimate the straight measure tail notch-lower tip jaw (right).

The curved shape of the animal’s position on the beach prevented an accurate measurement of the total (standard) body length. The measurement taken along the center of the animal’s body was 6.2 m (Fig 8). Another measurement, obtained from a drone photograph using a known person’s height (1.60 m) for scale, showed a straight distance between the median notch of the fluke (tail) and the tip of the upper jaw of 5.4 m (Fig 8). Thus, we estimate that the animal’s total length was approximately 5.8 m according to [6,37], the body size of the SRW calf at birth varies from 4.5 m to 6 m, i.e. on average 5.25 m. During the next 3–4 months, the calf grows in length at an average rate of 2.8 cm/day [6]. Considering the estimated body length of 5.8 m of the stranded specimen, the most likely postpartum time is then estimated at 20 days, with a minimum of zero (neonate) and a maximum of 46 days.

**Fig 9:**
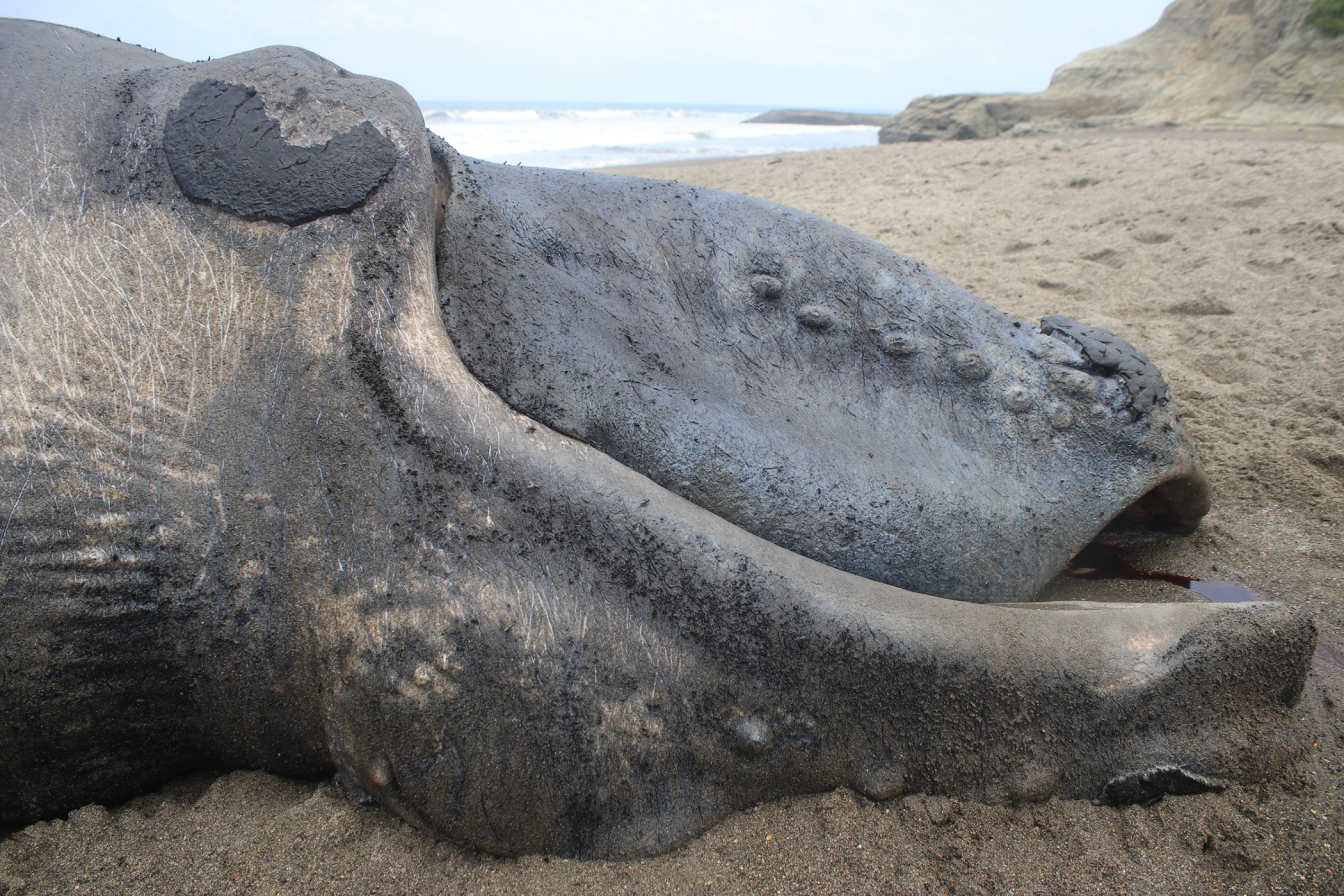
Left side of the head of the southern right whale calf stranded in Tongorachi, Ecuador, on 28 August 2023. Characteristics such as a strongly arched jawline, seven callosities on the lower jaw, and another three callosities on the maxillary, as well as linear wrinkle-like marks on top of and behind the blowhole, can be noted.

**Fig 10:**
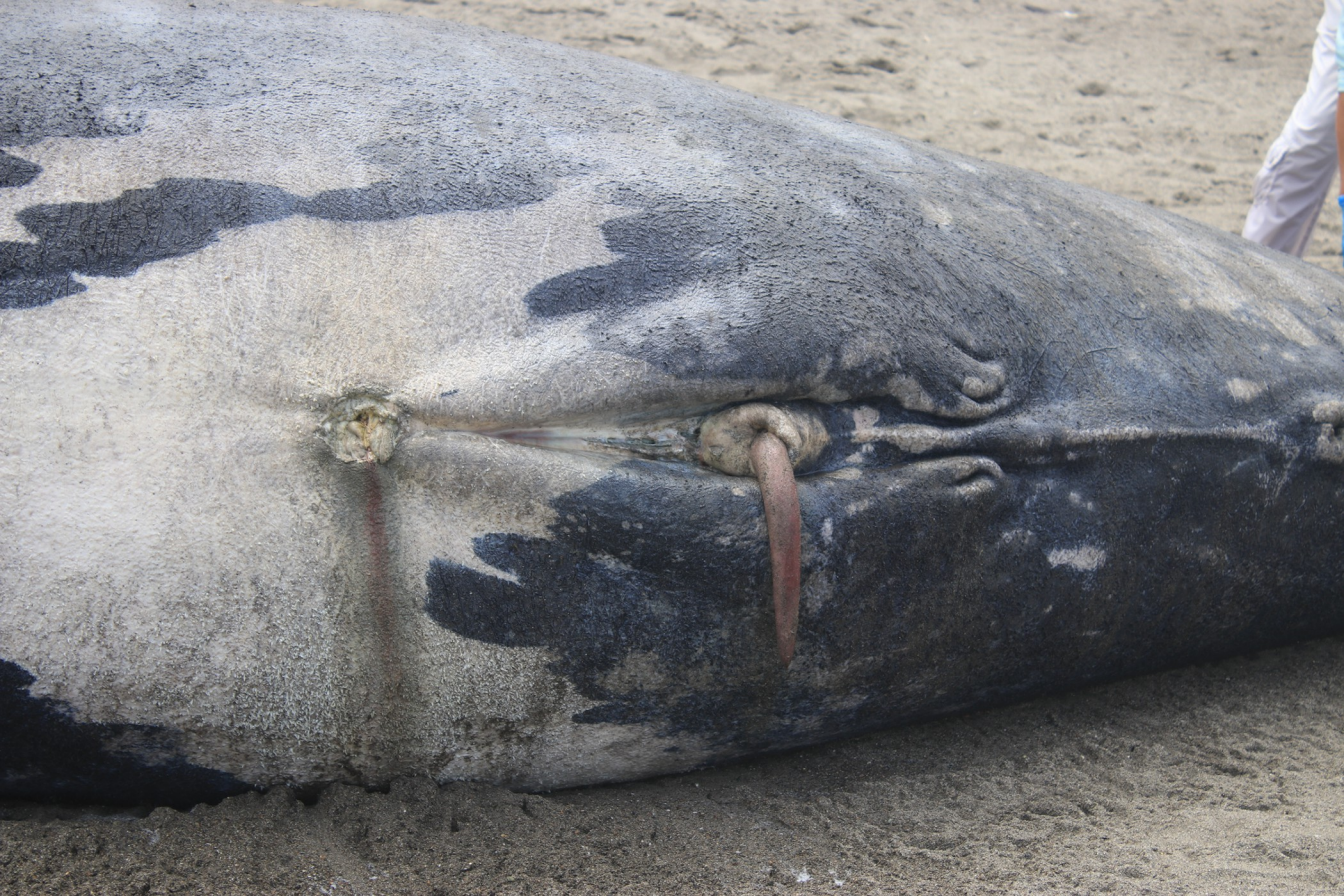
Ventral area of the southern right whale calf stranded in Tongorachi, Ecuador: Note asymmetrical white patches on the belly, umbilicus, genital slit with externally protruded penis, and lateral folds at the end of the genital slit.

The calf was dark-colored and displayed an asymmetrical and distinctive white pigmentation patch on its abdomen [38]. The mouth is prominently arched and began at the corner of the eye. The baleen has rudimentary, soft, pinkish-white baleen plates emerging from the upper jaw (Figs 8 and 9). Twelve slight creases on the left side between the eye and the blowhole. Seven prominent callosities were evident on either side of its lower jaw, with the bonnet formed by three callosities on the top of the head; additionally, a callosity was present on the lower jaw (Fig 9). The umbilicus was in the process of healing. The penis was red, measuring 35 cm, and was fully extended from the genital slit (Fig 10). The genital slit was elongated, originating from the umbilicus. Towards its distal end, the slit deepened, exhibiting two prominent folds on the sides and a central lineal protrusion (Fig 10). The specimen had tiny barnacles on the tail, peduncle and pectoral fins; no parasites were observed.

The external examination revealed parallel linear marks and cuts on the tail peduncle and fluke, suggestive of interactions with fishing gear. A 1 cm deep hole of unknown origin was observed on the upper right body. There was a laceration on the pectoral fin that could have occurred post-mortem. Samples of skin, blubber, cirripeds, and baleen were collected for further analysis. The cause of the stranding remained undetermined, but entanglement in fishing nets was suspected. Park rangers reported finding remnants of fishing nets nearby.

## Discussion

All records from Ecuador, Peru and Chile, presented herein, were authenticated with (S1 and S2 Videos) and photographs. They demonstrate external characteristics distinctive for SRW, including a very stocky body, absence of a dorsal fin, a strongly arched jawline and prominent callosities on the head [6,32,39]. The calf stranded revealed irregular white patches distributed over the posterior abdomen. Similar white markings have been observed in SRW individuals from South Georgia and South Africa [40], and are less common in northern relatives [39].

Our records expand the known northern distribution range of the SRW population in the ESP some 1,500 km from central Peru into northern Ecuador. The stranding at Esmeraldas, Ecuador, is also the first authenticated SRW record north of the equator and the world’s northernmost record of this species. Currents could have somewhat transported the carcass to the stranding site. However, given its fresh condition and the species’ very nearshore distribution, it is safe to presume that the calf and its mother were moving in the near vicinity of the stranding area. Preceding the above findings, both northernmost confirmed records of SRW mother-calf pairs were documented off metropolitan Lima, Peru; one on 21 August 2012, at Chorrillos (12°10’S) [17] and an earlier one on 30 July 2005 at Pucusana (12°29’S) [14]. The presence of calves among all the newly recorded sightings offers some optimism for the Critically Endangered ESP population, as successful reproduction stands is a promising indicator of population recovery. However, the calf found stranded dead in Tongorachi (Ecuador) highlights the ongoing threats to this population, such as interactions with fishing nets [19,20,38]. This incident underscores the need to continue and enhance monitoring and research efforts to better understand and mitigate the risks facing this population.

Three previous non-authenticated reports describe the possible earlier presence of SRW in Ecuadorian waters. The oldest claim is by Christensen [41], who noted in 1926 “that right whales occasionally visit the coasts of Ecuador, where they approach the sandbanks in winter”. Christensen was familiar with the area and conducted nine expeditions to the Southeast Pacific, starting in 1926 and continuing over the next ten years, exploring new hunting grounds and attempting to establish companies and factories for whaling and pearl harvesting between Ecuador, Peru and Chile [42]. Another report was made by Robert Clarke in 1965 [4], who suggested "SRW in some cases take advantage of the cold waters of California and the Humboldt Current to move into very low latitudes in the region of Ecuador". The third report is a probable sighting (supported by a low-resolution photograph) of two SRW off Punta Sal, Tumbes, in 2005 [14,43] but the species identification could not be fully confirmed. With the addition of these historical reports and multiple new authenticated Ecuadorian records, we suggest that this population be henceforth referred to by the name Eastern South Pacific (ESP) population, rather than the Chile-Peru (CPe) population.

Oceanographic conditions may play a pivotal role in shaping the migratory patterns of ESP right whales, notably in their extended presence towards northern regions. The Southeast Pacific region experiences a cyclical influence from the El Niño Southern Oscillation (ENSO) phenomenon, characterized by its warm phase (El Niño) and cold phase (La Niña) [44]. During the extreme El Niño event of 1982/1983, [45] noted a 65% decline in the abundance of blue (*Balaenoptera musculus)*, fin (*B*. *physalus)*, Bryde’s (*B*. *edeni)*, humpback, and sperm whales (*Physeter macrocephalus)* in northern Peru due to decreased primary productivity, while their numbers rose by 1.1% to 13% south of 8°S. Conversely, an abnormal increase in humpback whale catches off the central coast of Peru during the 1925/26 season was attributed to El Niño by [46], though without evidence of causality. Nonetheless, the unusually high numbers of nearshore (northbound) migrating humpback whales in central Peru during the 2023 El Niño [47] is consistent with Clarke’s hypothesis and seems to confirm an El Niño related effect, albeit for an unknown ecological reason.

During La Niña phases, SRW would venture further north perhaps driven by a stronger flow of the cold Humboldt Current [23]. Although specific data on whale range expansion in cold years within the ESP are lacking, correlations have been drawn between the presence of southern elephant seals (*Mirounga leonina*) in Ecuador and Panama with La Niña years [48,49] as lower sea surface temperatures resemble the seals’ normal habitat in cold austral waters. La Niña may also influence food availability in SRW sub-Antarctic feeding areas, although specific data are lacking. Antarctic ice cover and krill density are susceptible to temperature changes and affect prey availability for krill predators such as whales [50]. The reproductive success of Southwestern Atlantic right whales off Brazil is attributed to an inverse correlation between increases in krill density on the feeding grounds in South Georgia and the Oceanic El Niño Index (ONI) [51]. The recent reports of SRW mothers with calves in Ecuador in 2022 and 2023 would align with this pattern, but it could also be spurious (see below). Notably, between 2020 and March 2023, the ONI Index remained consistently negative, characteristic of the La Niña event, until sea surface temperature suddenly increased in the +1 and +4.5°C in July 2023, particularly off Ecuador and Peru [52].

### Potential range expansion

The first SRW record in Ecuador in 2022 coincided with La Niña event [22], but subsequent records, including the stranded calf, occurred during El Niño. The calf likely stranded shortly after birth, so it is likely that the mother-calf pair truly had reached the equatorial waters off the Esmeraldas Province. While there’s typically a nine-month lag in the response of right and humpback whales to increased krill density in feeding grounds [51,53], the temperature rose in the Southeast Pacific in February 2023 [54], preceding the whales’ migration to the breeding grounds. If the sea surface temperature was a decisive factor in SRW distribution, the whales might have avoided migrating so far north in 2023, similar to other baleen whale species during the El Niño of 1982 as reported by [45]. The lack of distributional change among SRW during El Niño events aligns with findings for humpback whales in Ecuador during the El Niño 1997–1998 [55], as both species do not typically feed in tropical regions. Decreased productivity impacts whale species feeding in lower latitudes, such as Bryde’s, blue, and sperm whales [45]. It could be suggested that the North Pacific right whale *Eubalaena japonica* (*Lacépède*, 1818) might be responsible for some of the Ecuadorian records, however this is exceedingly unlikely. Potential winter breeding grounds of *Eubalaena japonica* are thought to be located in latitudes 20°- 30°N. An exceptional (probably extralimital) record was registered off Baja California, Mexico, in February 1996 [39], a straight-line distance of 4,100 km from northern Ecuador. There are no right whale records for Central America. Moreover, the southernmost presence of *E*. *japonica* would imply a 6-month migratory phase difference with the SRW.

The apparent expansion of SRW distribution northward may be more plausibly linked to population recovery, akin to the expected resurgence of the South Atlantic right whale population towards northern Brazil [51]. Increased research efforts in the region will be important for a better understanding of the temporal variation in range and distribution of this population. Significant genetic differences have been identified among whale populations in Brazil and Chile-Peru, suggesting a more complex population structure [56]. New genetics and satellite tagging evidence demonstrate that Southeast Atlantic right whales may frequent the Southeast Pacific either because of population expansion or recolonization [57]. Thus, it is not discarded that some of our records in the Southeast Pacific correspond to Southwest Atlantic right whales [57].

Considering the broader ecological context, researchers have considered a potential influence of climate change on the observed range expansion of SRWs in the SW Atlantic [51]. Alterations in ocean temperatures and currents might be facilitating these whales’ movement into previously less frequented regions or where they were extirpated by whaling [51,56]. However, the three earlier reports, since 1926, of SRW occurring in ESP tropical waters [4,41,43], offer an alternative hypothesis, i.e. that SRW may always have visited northern Peru and Ecuador, perhaps in low numbers, but were not detected due to minimal historical sighting efforts and misidentification with sympatric humpback whales. Understanding the dynamic interplay between climate variations and potential range expansion is crucial in elucidating the ecological responses of SRW to environmental shifts. To gain deeper insights into the distribution and potential shifts of this population, ongoing cetacean monitoring in continental Ecuador and northern Peru, particularly during ENSO events, is essential.

### Acoustics

In the observation of northern Peru (Record #1, Table 1), the paucity of calls in the acoustic recording, despite proximity to the whales, is in line with what is known about right whale mother-calf pairs, *i*.*e*., acoustic crypsis [58,59]. This behavior consists of mother-calf pairs being largely silent, displaying low call rates, producing low-amplitude calls, or selecting acoustically isolated habitats, to avoid predator eavesdropping. The down-calls had lower frequencies and longer durations than those reported by [35] (Table 2). However, the values we reported here are based on a significantly smaller sample and are still within similar ranges, given that [35] reported large standard deviations for frequency and duration characteristics.

The presence of humpback whales in the study area, along with the detection of a single sequence of humpback whale song in the analyzed acoustic data, means that we cannot confidently rule out the possibility of that similar SRW calls are being mistaken for a humpback whale located further away. Most humpback whale songs exceed 500 Hz and typically do not resemble SRW calls (Fig 4); however, some sounds can occur below 100 Hz [60]. This is why we have classified these as ’possible’ SRW mother-calf down-calls.

### Conservation implications

The new records obtained of SRW are highly significant; however, their habitat, being so close to the coast, spatially coincides with anthropogenic activities including vessel traffic, fishing, unsustainable tourism, and marine pollution, all of which are considered potentially significant threats to the study population [61–64]. Marine traffic in both northern and southern Chile areas is experiencing intense growth due to expanding mining activities and maritime transport through the Strait of Magellan [61]. Mejillones Port (located 40 km from La Rinconada Marine Reserve, Chile) has previously been identified as a high-risk collision zone for humpback and southern right whales [61,62]. Several vessel collisions incidents have already been documented in southern Chile [20]. A near-collision with a purse-seine fishing vessel was observed near the Lima coast [14]. Southern right whale populations off South Africa and the eastern coast of South America (Brazil, Uruguay, and Argentina) suffer significant mortality due to vessel collisions [65]. At least four baleen whale species and sperm whales are impacted by these collisions in the Eastern Pacific [66]. Therefore, conservation efforts should focus on alerting vessels to the presence of whales and implementing vessel traffic management measures (e.g., real-time whale monitoring, speed restrictions, adaptation of shipping lanes) in areas where southern right whales concentrate.

According to [67], in Puerto López, the main whale-watching port in Ecuador, 77,625 visitors came to observe humpback whales in the marine area of Machalilla National Park (MNP) between June and September 2019. SRW including mother-calf pairs, have been observed navigating this area for two consecutive years, suggesting they may be re-establishing their presence, increasing their population, or moving to other areas. Regardless of the reason for these sightings, the high volume of tourism and vessels, coupled with minimally enforced regulations, could pose significant problems for the population. Given that these observations occur within a protected area, there is an opportunity to strengthen regulations for whale-watching vessels in this MNP to prevent disturbance and harassment of Critically Endangered SRW, especially mother-calf pairs. Continuous monitoring measures and training programs are crucial to promote sustainable ecotourism practices and environmental awareness among visitors. Furthermore, international cooperation in managing migratory routes and restoring marine habitats is essential for the long-term conservation of these vulnerable migratory species.

There is strong evidence of entanglement leading to mortality in ESP SRW. Historical stranding records dating back to 1986 [68], along with more recent cases in 2017 and 2023 [38,69] in Chile, underscore this issue. The stranding event in Ecuador further emphasizes the severity of the situation. Entanglement has been identified as a significant concern in the Conservation Management Plan (CMP) [70], and the IWC Scientific Committee has expressed serious concerns about anthropogenic mortality events related to entanglement.

Humpback whales serve as a valuable proxy for understanding the threats faced by SRW. Between 2001 and 2016 in Ecuador, 18 humpback whales were stranded with direct evidence of entanglement in fishing gear, accounting for 31% of cetacean strandings in the country [71]. This confirms that interaction with fishing nets is a major threat to cetaceans in Ecuadorian coastal waters. Such interactions could also impact SRW, given their sympatry with humpback whales during the same season [72]. Seasonal and specific modifications to fishing gear along the coastline should be implemented to reduce the risk of entanglement. Additionally, expanding or establishing marine protected areas or corridors is essential to safeguard critical habitats of SRW in Chile, Peru, and Ecuador. Promoting safe fishing technologies through education and training is also crucial. Regular monitoring and reporting of entanglement incidents and sightings by stakeholders are fundamental to enhance understanding and effectiveness of these measures and long-term conservation efforts for this population.

## Conclusion

Our study significantly extends the known range of the Southern Right Whale (SRW) in the Eastern South Pacific (ESP) population by approximately 1,500 km northwards to Ecuadorian waters. The authenticated stranding in Esmeraldas, Ecuador marks both the first confirmed SRW record north of the equator and the species’ northernmost global sighting. Additionally, Ecuador is now part of the distribution of this species with three consecutive records the last two years: two sightings and one stranding. Therefore, it is suggested that the sub-population should be recognized as the Eastern South Pacific southern right whale (ESP SRW). While La Niña conditions may be driving this change, the recent northward sightings could also indicate a positive response to ongoing population recovery efforts. However, the conservation outlook remains challenging due to anthropogenic threats such as vessel strikes, entanglement in fishing gear, and unsustainable tourism, all posing serious risks to this Critically Endangered population. Effective management strategies, increased monitoring, and continued research are essential to mitigate these threats and support the conservation of the ESP SRW population in their expanded range.

## Supporting information

S1 Video Southern right whale mother and calf observed off Salango Island, Ecuador. Note the arched shape of the mouthline.
(MP4)

S2 Video Southern right whale mother and calf observed off Sucre Islet, Ecuador. Note the absence of a dorsal fin and the presence of callosities on the head in the adult animal.
(MP4)
